# Endodontic management of a rare case of Type III Dens invaginatus in a maxillary canine combined with a previous occurrence of dental trauma

**DOI:** 10.4317/jced.61467

**Published:** 2024-06-01

**Authors:** Giorgos N. Tzanetakis, Eleni Mougiou, Despina Koletsi, Nikos N. Lygidakis

**Affiliations:** 1 DDS, MSc, MSc, PhD. Associate Professor, Department of Endodontics, School of Dentistry, National and Kapodistrian University of Athens, Athens, Greece; 2 DDS. Private Dental Practice, Athens, Greece; 3 DDS, MSc, Dr. med. dent, MSc DLSHTM, PGCHEd. Senior Teaching and Research Staff, Clinic of Orthodontics and Paediatric Dentistry, Center of Dental Medicine, University of Zurich, Switzerland; 4 DDS, MSc, Dr. med. dent, MSc DLSHTM, PGCHEd. Research Affiliate, Meta-Research Innovation Center at Stanford (METRICS), Stanford University, California, USA; 5 BDS, MPaedDentRCS (Engl), DrDent (UCL). Paediatric Dentist, Private Paediatric Dental Clinic, Athens, Greece

## Abstract

Dens Invaginatus (DI) is a developmental anomaly which eventually leads to pulp necrosis and has several clinical implications in sufficient instrumentation and obturation of the root canal system. The present clinical report presents a rare case of a maxillary canine affected with DI leading to pulp necrosis combined with a previous dental trauma, which also led to irreversible pulp damage of the adjacent lateral incisor. A 14-year-old male patient with a history of dental trauma at the right maxillary region, one year earlier, was referred with pain and swelling at the apical area of the right maxillary canine. After CBCT evaluation, complete removal of the invagination was decided. All the procedures were performed under operating microscope and canal obturation was done with apical plug technique using MTA. Two-year follow-up radiographic assessment confirmed complete healing of the periapical tissues for both teeth. The present case describes a rare case of dens invaginatus in a maxillary canine pointing out the importance of obtaining a thorough dental history upon diagnosis, performing also a careful clinical and radiographic evaluation and subsequent treatment planning, especially when addressing complex pathologies and unusual dental malformations. In such cases with high degree of complexity, preoperative CBCT examination is required for decision making and subsequent appropriate management.

** Key words:**Dens Invaginatus, maxillary canine, endodontic treatment.

## Introduction

Dens Invaginatus (DI) is a developmental dental anomaly that occurs prior to calcification of dental tissues, due to the invagination of the enamel organ into the dental papilla (1,2). Its etiology has not been fully elucidated; several theories have been considered regarding etiopathogenesis, while genetic or epigenetic factors such as infection or dental trauma cannot be precluded (1,2). This type of malformation has two main clinical implications, first, it leads to pulp necrosis due to bacterial infection through the portal of invagination and second, the unusual morphology creates major difficulties to appropriate chemomechanical preparation and sufficient root canal obturation (3,4).

The prevalence of permanent teeth affected with DI has been reported to range from 0.3% to 10%2. Two recent systematic reviews (SRs) reported that the occurrence of DI is not as uncommon as it has been formerly believed (5,6). The most updated of these SRs reported a prevalence of 7.45% among 7373 individuals5. On tooth level, the respective prevalence was reported to be 0.72%5. It should be noted that five out of the six studies included patients originated from Asia (7-11), whereas the sixth included Tunisian subjects (12). Some other prevalence studies not included in the above meta-analyses have also been conducted in Turkish derived individuals (13-15). Regarding tooth type, maxillary lateral incisors are the most frequently affected teeth, followed by maxillary central incisors, maxillary canines, and premolars, revealing that maxillary arch is affected more often compared to mandibular one (5-9).

The occurrence of DI in maxillary canines is generally uncommon. Capar *et al*. showed that the prevalence of DI in maxillary canines in a Turkish population is 4.25% (7). This is the highest percentage of prevalence that has been ever reported in maxillary canines. However, a similar study again in Turkish population detected a significantly lower prevalence of 0.02%. More recently, Varun *et al*. (9) concluded that the same prevalence in an Indian population was 2.95%. No evidence exists on the recording of differential distribution of DI prevalence in maxillary canines across population groups geographically dispersed; that is possibly due to diverse genetic and epigenetic effects acting in different races and populations. The latest meta-analysis on this topic revealed a global prevalence of 0.01% for DI in maxillary canines, confirming the rareness of the defect (5).

Only fourteen cases have been described so far in the endodontic literature (16-28). These cases have been endodontically treated either conventionally or both conventionally and surgically ( Table 1). in most of the cases, conventional management led to successful treatment overall. Despite the major difficulties that may be encountered during the chemomechanical preparation and the obturation of such root canal systems, it appears that conventional endodontic approach is highly effective.

The present clinical report describes the endodontic management of a rare case of a maxillary canine affected by DI and presented with pulp necrosis, combined with a previous dental trauma which led to irreversible pulpal damage of the adjacent lateral incisor making the diagnosis of the case more complicated, and the management more complex and sophisticated.

## Case Report

**Figure 1:**
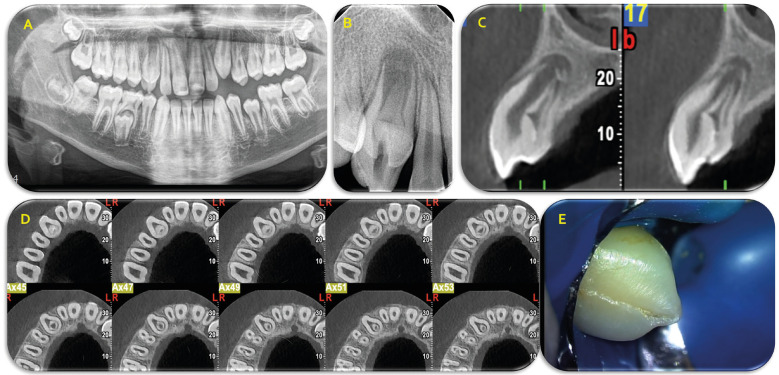
A. Panoramic radiograph of the patient revealing the right maxillary canine with the invagination, B. Initial periapical radiograph of the case at the time of referral, C, D. Sagittal and axial CBCT scans showing the relation of the invagination with the root canal walls, E. Clinical figure of the buccal surface of the crown of the tooth.

A 14-year-old male patient with a non-contributory medical history, but with history of dental trauma one year earlier at the right maxillary region was referred for root canal treatment on the right maxillary canine. The young patient had already sought emergency dental treatment for an acute apical abscess on the canine by visiting his pediatric dentist who endodontically accessed the root canal to alleviate the symptoms. The referring dentist had already observed the existence of dens invaginatus in the canine though a panoramic radiograph (Fig. 1A) and before the referral he had prescribed a CBCT examination (Fig. 1C,D).

**Figure 2:**
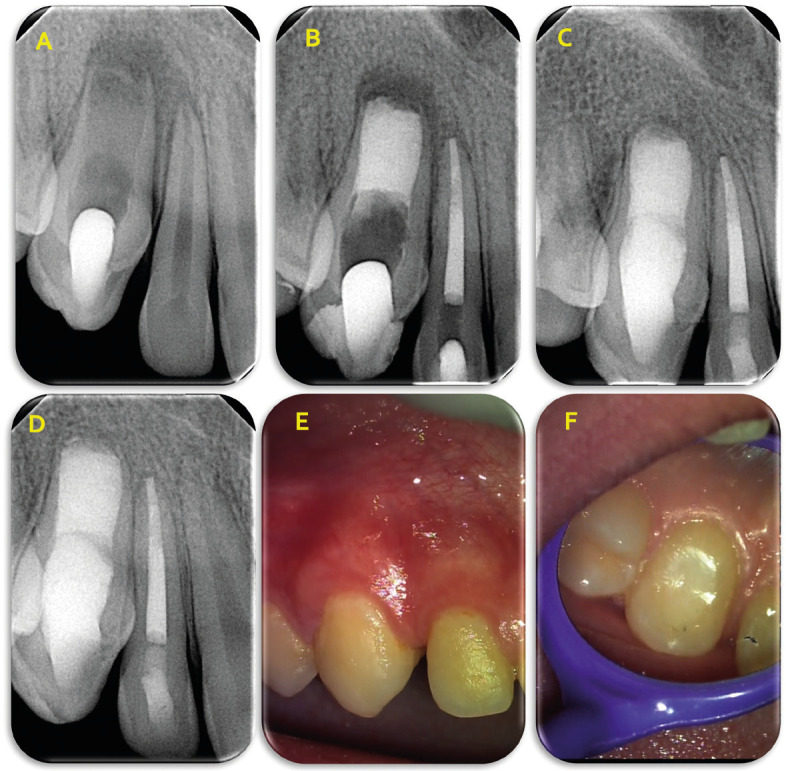
A. Periapical radiograph confirming the complete removal of the invagination, B. Final radiograph of the case, C. 1st follow-up radiograph at 12 months D. 2nd follow-up radiograph at 24 months, E,F. Clinical appearance of the teeth and the soft tissues during second recall examination.

When the patient proceeded to the endodontist’s office, he was pain free, but the apical swelling had not completely subsided. Due to previous access preparation of the canine as it is shown in the initial periapical radiograph (Fig. 1B), pulp sensibility tests were performed only on the adjacent teeth, with the left maxillary lateral incisor revealing a quite delayed response to electric pulp testing (EPT) and no response to cold test. For all treatment procedures that will be described subsequently an informed consent was obtained by the parents of the young patient. At first place, it was decided to complete the endodontic treatment of the maxillary canine and to repeat pulp sensibility tests on the lateral incisor at the next appointment. However, CBCT evaluation led us to the decision to completely remove the invagination due to morphological characteristics of the case because the invagination was attached only onto the cervical root canal walls. In addition, the anticipated difficulties that could be encountered towards the achievement of a satisfactory chemomechanical preparation and adequate obturation of the root canal system in the presence of the invagination dictated the aforementioned treatment plan. The tooth was initially anesthetized with a buccal and palatal infiltration of articaine with epinephrine as vasoconstrictor 1/100:000 (Orablock, Pierrel S.p.A, Capua, Italy) and isolated with a rubber dam (Fig. 1E). Following the improvement of access cavity preparation, root canal system was irrigated with copious amounts of NaOCl 2.5%. The removal of the invagination was performed using Munce micro low-speed bars initially (Munce Discovery bars, CJM engineering endodontic technologies, Ojai, California, USA), and ultrasonic U-files No 25 at the final stages of the removal. A periapical radiograph was taken to confirm the entire removal of the invagination (Fig. 2A). All the procedures were performed under operating microscope at magnification 12.5x (OPMI PICO, Carl Zeiss Meditec AG). Following this procedure, root canal was irrigated again with NaOCl 2.5%. Final irrigation was performed by using EDTA 17% for smear layer removal. Mechanical instrumentation was kept to a minimum due to root canal width. Only circumferential filing was performed and finally ultrasonic activation of NaOCl took place, using U-files No 15 for improved disinfection of the root canal system. The canal was dried with paper points and dressed with pure calcium hydroxide mixed with sterile saline solution.

Two weeks later, the canine was completely asymptomatic with no tenderness to percussion, however, a redness of the soft tissues apically of the lateral incisor was evident. Pulp sensibility tests were repeated with no response to cold and electric pulp testing. A diagnosis of pulp necrosis was set for the maxillary lateral incisor. Thus, at that second appointment, besides the canal obturation of the canine, endodontic treatment of the lateral maxillary incisor took place as well. Root canal instrumentation of the incisor was performed by using Edge X7 rotary NiTi files (Edge Endo) with the final file being No 35/4%. Root canal was irrigated with NaOCl 2.5% and EDTA 17%. Canal obturation was done using vertical compaction of warm gutta-percha.

Root canal obturation of the canine was performed with apical plug technique using MTA (Fig. 2B). MTA was placed up to the middle of the canal without placing gutta-percha on top of MTA. The remaining canal space was scheduled to be filled with composite resin by the referring dentist. No collagen sponge was used during the process. The access cavity was temporarily sealed with Cavit G and the patient was referred to his pediatric dentist for permanent restoration and was scheduled for a close recall monitoring.

-Follow-up examination

The 12-month follow-up radiograph showed complete healing of the periapical tissues of both teeth, while their crowns were restored with composite resin by the referring paediatric dentist (Fig. 2C). The 2-years clinical and radiographic reevaluation of the patient confirmed the favorable healing of the tissues (soft and hard periapical) with no any adverse events (Fig. 2D,E,F).

## Discussion

The present report describes the endodontic management of a maxillary canine with Oehlers type III of DI associated with pulp necrosis and acute apical abscess. In the present case, the co- existence of previous traumatic injury in the area of interest made the final diagnosis complicated and the treatment planning utterly challenging. The endodontic management involved the adjacent lateral incisor as well, leading eventually to dissolution of clinical symptoms and complete healing of the periapical tissues of both teeth. The case demonstrates the importance of obtaining an accurate dental history report before any clinical action takes place for the management of such teeth with multiple pathologies.

A thorough search of the literature revealed that the present case constitutes only the fifteenth case described globally and being endodontically managed successfully ( Table 1). Three more cases have been described (29-31), however, two teeth were extracted, the first due to a multidisciplinary approach followed that was judged more beneficial for the young patient (29), and the second due to misdiagnosis of DI as an odontome (30) whereas the third was managed unsuccessfully using only a surgical approach (31). The case with the misdiagnosis30 evidently reveals the importance of preoperative CBCT evaluation in precise and accurate interpretation and decision making in similar cases with unusual canal system morphologies and subsequently in adequate treatment planning and appropriate clinical management.

Several treatment approaches have been suggested for the management of DI especially with Oehlers type III malformation. Most cases have been managed by implementing a conventional approach with warm vertical condensation of gutta-percha as root canal obturation technique. It is worth noting that few cases of DI have been successfully managed so far by using a regenerative procedure (27,32,33). In these reports, the authors justified their preferred approach on the grounds of complexity and maturation of the invaginated canine. CBCT preoperative evaluation was crucial towards final decision making.

The approach of the removal of the invagination followed in the present case has been previously described for the management of DI in two maxillary lateral incisors (33,34). The same approach was also followed by Ciracoglu *et al*. (28) in a case of a maxillary canine like the present. The decision to completely remove the invagination was directly driven following preoperative CBCT evaluation and the use of operating microspore under high magnification. In the present case, this was considered feasible due to the loose type of attachment of the invagination onto the root canal walls, as it was revealed by the evaluation of axial and sagittal CBCT scans. The advantage of this approach is the anticipated improved disinfection outcome of the root canal system, compared to a system with the invagination area present along with narrow isthmuses and small ramifications, that are entities difficult to be accessed by mechanical instrumentation and disinfection agents. Thus, it may be speculated that when the internal morphology allows the complete removal of the invagination, it would be preferential to proceed with an approach that minimizes the infection potential of the tooth.

One might argue that the removal of the invagination may lead to reduced mechanical resistance of the affected tooth. However, in the present case, and considering the axial CBCT scans, it was evident that the root canal walls of the canine were apparently thick enough to allow for such an approach, with a potential exception of the apical third area, which demonstrated an appearance of immature tooth. To further add, the main mass of the invagination appears at the cervical and middle third of the root, and this allowed us to remove the invagination in a predictable manner, without mechanically impacting further the main root canal walls of the tooth.

MTA apical plug technique was opted for canal obturation in our case. This was done since the technique is reported to provide high success rates and is considered more established and predicTable compared to the regenerative endodontic procedure (35). As in the removal of the invagination, the placement of MTA was performed under high magnification, to achieve a clearly visible apical area during the compaction of the material. MTA was placed until the middle of the canal to avoid any possibility of crown discoloration in the future. In this respect, the placement of permanent restorative material was of utmost importance and subsequently provided a hermetic seal of the access cavity. In the present case, despite the extended depth of the cavity, the referral pediatric dentist accomplished the final restoration in close proximity to MTA. The latter is considered important to avoid undesirable voids between the MTA and the permanent restorative material.

In conclusion, we may suggest that similar cases of DI, even in teeth with exceptionally low prevalence, should receive a personalized individual assessment, to reach an accurate diagnosis and decide the appropriate management. The latter seems to be grounded largely on the precise preoperative CBCT evaluation, which will always reveal the variety of treatment alternatives for the management of such unusual canal system morphologies.

## Figures and Tables

**Table 1:** All published cases of maxillary canines with Dens Invaginatus that have been endodontically managed along with the characteristics of each case.

Case	Ref	Age / Gender	Continent	Clinical signs / Symptoms	Preoperative pulp status	CBCT preop evaluation	Preoperative Periapical diagnosis	Management	Outcome
1	(16)	13y / F	UK	Sinus tract	MC: Vital pulp DI: Pulp necrosis	No	Chronic apical abscess	Combined (conventional and surgical)	Successful (no follow-up radiograph provided
2	(16)	14y / M	UK	Intraoral swelling	Not mentioned	No	Acute apical abscess	Surgical	Successful (no follow-up radiograph provided
3	(17)	11y / M	USA (Caucasian)	Pain, Swelling,	Pulp necrosis	No	Periapical lesion	Conventional	Successful
4	(18)	30y / F	Canada	Sinus tract, extreme sensitivity to cold	MC: Symptomatic, Irreversible pulpitis DI: Pulp necrosis	No	Chronic apical abscess	Combined (conventional and surgical) (Re-surgery after 7 months)	Initially unsuccessful Successful after re-surgery
5	(19)	14y/ M	Israel	Intraoral swelling	Not mentioned due to previous access before the referral	No	Chronic apical abscess	Conventional	Successful
6	(20)	16y / F	USA	Throbbing pain	MC: Vital pulp DI: Pulp necrosis	No	Chronic apical abscess (associated only with the invagination)	Conventional (only the invagination)	Successful
7	(21)	18y / F	Poland	None at the time of referral	Pulp necrosis	No	Chronic apical abscess	Conventional	Successful
8	(22)	13y / F	Turkey	Spontaneous pain	Pulp necrosis	No	Symptomatic apical periodontitis	Conventional	Successful
9	(23)	12y / M	Iran	Sinus tract	MC: Vital pulp DI: Pulp necrosis	No	Chronic apical abscess (associated only with the invagination)	Conventional	Successful
10	(24)	13y / F	Spain	Pain, Swelling	MC: Vital pulp DI: Pulp necrosis	Yes	Acute apical abscess	Conventional (Obturation of the invagination with MTA)	Successful
11	(25)	14y / F	USA	Sinus tract	MC: Vital pulp DI: Pulp necrosis	No	Chronic apical abscess (associated only with the invagination)	Conventional (entire root canal system)	Successful
12	(26)	17y / F	India	Sinus tract	Pulp necrosis	Yes	Chronic apical abscess	Conventional	Successful
13	(27)	11y / M	Korea	Pain, Swelling	Pulp necrosis	Yes	Chronic periapical abscess	Regenerative	Successful
14	(28)	40y / F	Turkey	Pain, Swelling	Pulp necrosis	Yes	Symptomatic apical periodontitis	Conventional (Complete removal of the invagination)	Successful

mo, months; y, years; MC, main canal; DI, Dens Invaginatus;

## Data Availability

The datasets used and/or analyzed during the current study are available from the corresponding author.
